# Use of dried blood for measurement of trans fatty acids

**DOI:** 10.1186/1475-2891-8-35

**Published:** 2009-07-24

**Authors:** Ruby Gupta, Ransi Ann Abraham, Savita Dhatwalia, Lakshmy Ramakrishnan, Dorairaj Prabhakaran, Kolli Srinath Reddy

**Affiliations:** 1 Department of Cardiac Biochemistry, All India Institute of Medical Sciences, Ansari Nagar, New Delhi-110029, India; 2 Center for Chronic Disease Control, Safdarjung Development Area, New Delhi-110016, India; 3 Public Health Foundation of India, New Delhi-110016, India

## Abstract

**Background:**

Fatty acid measurements especially trans fatty acid has gained interest in recent times. Among the various available biomarkers, adipose tissue is considered to be the best for the long term dietary intake but the invasive nature of tissue aspiration reduces its utility. Phlebotomy is a much less invasive method of sample collection when a large number of participants are involved in the study and therefore is an alternative, most suitable for large population based studies. In the present study fatty acid (with special emphasis on trans fatty acid) extraction from blood spotted and dried on filter paper was carried out to simplify the sample collection procedure and transportation.

**Methods:**

Blood samples were collected from 19 healthy volunteers. The blood was spotted (30 spots of 10 μl each) on filter paper, dried at room temperature and stored at 4°C in zip-lock poly bags. For comparison whole blood stored at -70°C was simultaneously analyzed.

**Results:**

A good agreement was seen between trans fatty acid values obtained in dried blood and whole blood as evident from the pearson correlation coefficients ('r' for monounsaturated (trans) 0.70 and for polyunsaturated (trans) 0.692 respectively). The intraclass correlation coefficient for monounsaturated trans was 0.805 and for polyunsarurated trans was 0.776.

**Conclusion:**

Dried blood spots can be used for trans fatty acid analysis.

## Background

Fatty acid measurement in adipose tissue, whole blood, erythrocyte membrane, serum or plasma, and specific plasma fractions serve as biomarkers of exogenously consumed fatty acids [1,2]. The different biomarkers reflect intake over several hours to past few years. Adipose tissue is considered to be the best biomarker for the long term dietary intake [3] because of its slow turnover but the invasive nature of tissue aspiration reduces its utility. Whole blood as a biomarker of fatty acid assessment is amenable to widespread usage in epidemiological studies due to the relative ease of collection, processing and storage compared to other biomarkers. Fasting whole blood has been reported to be a suitable biomarker, with performance comparable to that of fasting plasma [4].

Measurement of trans fatty acids intake is of interest due to adverse health implications [5-7]. A suitable biomarker for trans fatty acid which is relatively less invasive and easy to transport would be useful in epidemiological studies where a central laboratory is responsible for analysis from blood collected from far off areas. Blood samples collected in the field needs to be stored at very low temperatures and transported in dry ice for measurement of fatty acids. A method that circumvents the need for blood processing, storage and shipment of samples at very low temperatures would therefore be desirable and dried blood is a promising alternative. The filter paper matrix stabilizes most analytes in dried blood spots, but the rate of sample degradation will vary from analyte to analyte and storage conditions. In the present study we explored the possibility of blood collected on filter paper and stored at 4°C for measurement of fatty acids including trans fatty acids.

## Method

Fasting blood samples were collected from 19 volunteers who were apparently healthy. Written consent was obtained from all volunteers. 5 ml of blood was collected in vacuum tubes with EDTA as additive. Replicates of blood spots (30 spots of 10 μl each) were prepared by dropping blood on Whatman filter paper (no. 3) kept on a non absorbent surface and allowed to dry at room temperature, transferred to a zip-lock poly bag and stored in a refrigerator (2–8°C ) for fifteen days. Whole blood was stored at -70°C till the time of analysis. Fatty acids were analyzed in both the dried blood and the corresponding whole blood samples. For fatty acid analysis in dried blood, ten punches of dried blood spot of 6 mm size each were taken in culture tubes with Teflon caps. 200 μl saline was added to extract blood from the filter paper. For whole blood fatty acid estimation 200 μl whole blood was directly used for fat extraction. 1800 μl of isopropanol/chloroform (11:7, v/v) containing 50 mg of 2,6-di-tert-butyl-p-cresol as an antioxidant was added to the extract from dried blood and whole blood samples [8]. The tubes were left at room temperature for an hour with intermittent shaking. The lower chloroform layer was aspirated and dried under nitrogen. Esterification was done using method of Leepage & Roy [9] with modification. 500 μl methanol-acetyl chloride 20:1 (v/v) was added to the tubes and heated in water-bath at 100°C for 1 hour. 3 ml of cold potassium carbonate was added to each tube slowly with continuous shaking. Methyl esters were extracted with 200 μl hexane. The solvent was evaporated under the stream of nitrogen and esters were re-dissolved in iso-octane. 1 μl sample was loaded on fused silica capillary cis/trans column SP 2560, 100 m × 250 μm internal diameters × 0.20 μm film (Supelco, Belefonte, Pennsylvania). The port temperatures of both the injector and the detector were set at 250°C. The oven temperature was initially set at 90°C for 4 min and was then increased 15°C/min until a temperature of 150°C was reached and held for 10 min, the temperature was further increased at 1°C/min till 170°C after which rate of temperature change was 5°C/min until a temperature of 230°C was reached and maintained for 30 minutes. The total run time was 80 min. A split ratio of 1:10 and an injection volume of 1 μl were used. The Gas Chromatograph was equipped with Flame – ionization detector using Nitrogen as carrier gas. Sample fatty acid methyl ester peaks were identified by comparing their retention times with those of known standards (Fatty Acid Methyl Esters from SUPELCO). Each peak was quantified by calculating the area under the peak using software from AIMIL (Nucon Technologies). The concentration of individual fatty acid was expressed as percentage of total area under the peaks.

Extraction and methylation for whole blood and their spots were performed in pairs to avoid within pair variability. C 17:0 was used as an internal standard. All reagents used were of HPLC grade. Known standards of fatty acids: a mixture of 37 components (C4–C24), and individual trans fatty acid methyl esters: Palmitelaidic acid methyl ester (C 16:1, 7t), Elaidic acid methyl ester (C18:1, 9t), Vaccenic acid methyl ester (C18:1, 11t), Linoleic acid methyl ester isomer mix (C 18:2, 9,12 – tt, tc, ct & cc) were run for identifying peak retention time of individual fatty acids in unknown samples. Monounsaturated, polyunsaturated, saturated and trans fatty acids (MUFA and PUFA) total were recalculated from the sums of fatty acids detected in each category. The fatty acids included among total saturated fatty acids are C 12:0,C 14:0,C 16:0 and C 18.0, and those in total monounsaturated (cis) are C 14:1 cis, C 16:1 cis and C 18:1 cis. Total PUFA (cis) includes C 18:2 cis, cis, C 18:3 n-6, C 18:3 n-3 and C 20:4 n-6. Total MUFA (trans) comprises of C 16:1 7-trans, C 18:1 9-trans and C 18:1 11-trans. The total PUFA (trans) included C 18:2 trans, trans, C 18:2 trans, cis and C 18:2 cis, trans.

Pearson correlation and Intraclass correlations were calculated to determine correlation between values obtained with dried blood and whole blood. Data analysis was done with SPSS software package.

## Results

**Figure 1:**
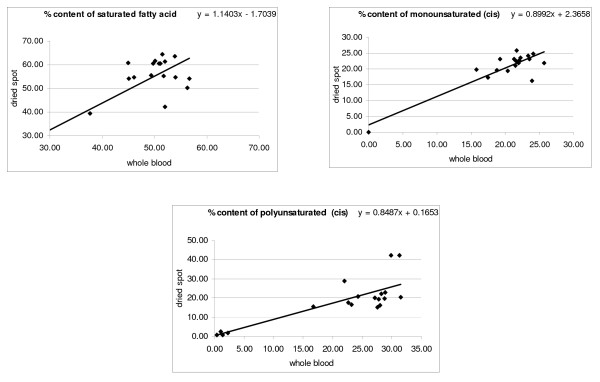
**Scatter-plot for saturated, mono unsaturated (cis) and poly unsaturated (cis) fatty acid extracted from whole blood and dried blood spot**. Values are percentage of total fatty acids.

**Figure 2:**
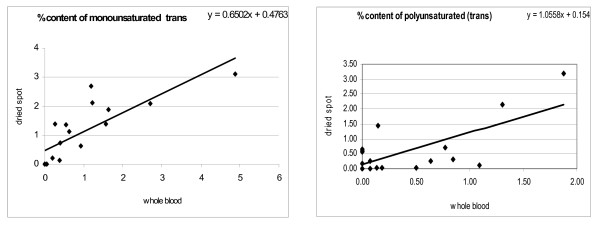
**Scatter-plot for mono unsaturated (trans) and poly unsaturated (trans) fatty acid extracted from whole blood and dried blood spot**. Values are percentage of total fatty acids.

Coefficient of variation for measurement of fatty acids when same sample was run in triplicates was 1.05% for saturated fatty acid, 0.77% for mono unsaturated fatty acid, 1.03% for poly unsaturated fatty acid and 6.55 for total trans fatty acid. For Inter day precision, same sample was run on six different days and the coefficient of variation for saturated fatty acid was 1.52%, for monounsaturated it was 1.55%, for polyunsaturated it was 0.66% and for total trans it was 13.25%. Figure 1 & Figure 2 shows the correlation between fatty acids in individual whole blood samples as against dried blood samples. As is evident a good agreement was found between saturated, MUFA and PUFA values obtained with dried blood and whole blood. The correlations coefficient (r) for trans MUFA and PUFA were 0.70 and 0.69 respectively. The trans fatty acid content was low in most of the samples. The Intraclass Correlation Coefficient for monounsaturated and polyunsaturated trans was 0.804 and 0.776 respectively, whereas for cis it was 0.890 and 0.937 respectively. The ICC for saturated fatty acid was 0.880 (Table 1).

**Table 1:** Correlation between fatty acids extracted from whole blood and dried blood spots (n = 19)

	**Pearson correlation coefficient 'r'**	**Intraclass correlation coefficient** **(95% CI)**
**Total saturated fatty acid**	0.827	0.880 (0.688 – 0.954)

**Total monounsaturated fatty acid (cis)**	0.883	0.937 (0.837 – 0.976)

**Total polyunsaturated fatty acid (cis)**	0.804	0.890 (0.715 – 0.957)

**Total monounsaturated fatty acid (trans)**	0.701	0.805 (0.493 – 0.925)

**Total polyunsaturated fatty acid (trans)**	0.692	0.776 (0.418 – 0.913)

## Discussion

Fatty acid analysis from dried blood has been previously reported in the diagnosis of Adrenoleukodystrophy [10,11]. These studies did not look at stability of fatty acids in dried blood when compared with whole blood. The comparison of blood spot assay results with those from matched, simultaneously collected fresh samples through venipuncture is a validation tool to assess suitability of dried blood for analysis of specific analytes. The present study shows a good correlation between dried blood and fresh whole blood with correlation coefficients ranging from 0.70 to 0.88 (Figure 1 &2).

The previously reported papers have also not looked at trans fatty acids in dried blood. Analysis of trans fatty acids has generated considerable interest in developing countries specially India where the prevalence of coronary artery disease is on the rise and the traditional risk factors are not able to explain the high rate of premature CAD in the country. Since the eating habits of Indian differ considerably from the Caucasians with consumption of more oil and fried food, trans fatty acid may be an important dietary contributing factor in predisposing Indians to CAD. Studies are needed in India to address this issue but the invasive nature of adipose tissue collection which is a gold standard for dietary fat intake is an obstacle in planning large population based studies. Blood may be an ideal choice but transportation of blood samples at very low temperatures to central laboratories for trans fatty acid estimation may be an issue. In this regard dried blood may be a viable alternative. In the present study we have demonstrated that trans fatty acids are stable in dried blood up to 15 days when stored at 4°C. This will make transportation easier. In conclusion dried blood spots can be used for trans fatty acid analysis. Collection of blood spots eliminates the procedures of centrifugation and transportation of samples in dry-ice. This has an application in multicentric studies where large number of samples is involved.

## Abbreviations

PUFA: Polyunsaturated fatty acid; MUFA: Monounsaturated fatty acid; ICC: Intraclass Correlation Coefficient.

## Competing interests

The authors declare that they have no competing interests.

## Authors' contributions

RL conceived the study design and wrote the manuscript, RG and SD carried out the work and KSR, DP & RAA provided the intellectual input for manuscript preparation. All authors read and approved the final manuscript.
